# Personal

**Published:** 1915-07

**Authors:** 


### PERSONAL.

**Announcement of removal, travel, and other matters of interest are requested. Please report errors in the listing of any physician in the State and other directories, that we may co-operate with the State Society in securing a correct list.**

---

The automobile of Dr. L. M. Wilkins of Lackawanna was stolen June 6 and was abandoned on the tracks of the Buffalo Creek R. R. Co., undamaged. He was slightly injured in an auto collision, June 14.

---

Dr. Nelson W. Strohm of Buffalo announces the removal of his office to 56 Allen Street.

---

Congratulations are due our Associate Editor, Dr. Charles Haase of Elmira. He was married, June 1 to Miss Helen Margaret Marx of Binghamton.

---

Captain Harry R. Trick of Buffalo spent the latter half of May touring in New England.

---

Dr. Eli G. Jones, Dartmouth, 1871, formerly of Burlington, N. J., resides at 897 West Ferry Street, Buffalo.

---

Dr. Charles J. Rosengren of Buffalo is taking a vacation of two months, during which he will visit San Francisco.

---

Dr. A. L. Benedict of Buffalo visited Atlantic City, staying at the Chalfonte, New York and Western Connecticut late in May and early in June.

---

Dr. Frank B. Willard of Buffalo has been promoted from Sanitary Inspector to Medical Sanitary Inspector, in the Buffalo Health Department. Dr. Alfred Regan has had a similar promotion.

---

Dr. John M. Swan of Rochester has been elected secretary of the American Society of Tropical Medicine, which met in San Francisco in June.

---

Dr. Charles A. Aaron of Detroit visited friends in Buffalo, June 12 and 13.

---

Dr. Lucien Howe of Buffalo attended the commencement of Bowdoin College at Brunswick, Me., and the meeting of the

American Ophthalmologic Society at New London, Conn., late in June.

---

Dr. Ray H. Johnson of Buffalo was burglarized in the night of June 21-22, losing over $400 in money besides watches and valuables.

---

Dr. Grover W. Wende, of Buffalo, retiring president of the Medical Society of the State of New York, gave a dinner in honor of Dr. A. T. Lytle at the Saturn Club, June 23. Covers were placed for 23, the guests being members of the committee of entertainment of the Society at its annual meeting in Buffalo. In token of his sacrifice of time and convenience for a period of many months in assuring a successful meeting, the members of the committee united in presenting to Dr. Lytle a solid silver tea service.

---

Dr. Herbert E. Baright has opened at Saratoga Springs a sanitarium for the treatment of gastro-intestinal, nutritional, and related disorders.

